# No “Battery” in a Tooth

**Published:** 1880-06

**Authors:** Chas. Mayr

**Affiliations:** Springfield, Mass.


ARTICLE III.

### NO "BATTERY" IN A TOOTH.

BY CHAS. MAYR, A. M., SPRINGFIELD, MASS.

A new opinion advanced with the emphasis, with which Dr. H. S. Chase defends the hypothesis of galvanism in teeth filled with gold, cannot fail to attract general attention. In a club of dentists of this city my attention as a specialist in practical chemistry and electricity was drawn to this subject and chiefly to article IV in the "Independent Practitioner" of February 1880, pages 77-81.

When I had read the article, I felt my whole chemistry and electricity crumble to pieces, if the facts at the bottom of page 79 were true; they were so startling, that I at once began careful investigations, and the results were so completely in accordance with the well known facts of chemistry and so absolutely against Dr. H. S. Chase's "facts," that in the interest of truth and science I thought it necessary to give my results here.

I first have to correct some erroneous statements of Dr. Chase, so discordant with the "text books on electricity," that "every dentist who has studied the elements of electrical science" must admit their untenability.

Dr. Chase always uses the term "battery" instead of element or combination; a battery is a combination of *several* cells, each cell containing an electric combination, or of elements; a tooth never can be a battery, it can only be a cell or a combination, as long as it does not contain several distinct cavities all properly prepared and connected.

Then he says: "A galvanic battery may be composed of almost any two different substances." The little word "almost" is very important. Why not of any? If Dr. Chase goes further in his "text books" he will find that *both* substances must be conductors of electricity. Thus we have electrical combinations not only of two metals, but also of two liquids, even of metals and salts, but always both substances must be conductors; e. g. glass at common temperature is a non-conductor of electricity, at least not a worse conductor than dry dentine or "dentos," and no "battery" can be made in which glass is one element, even if the liquid be hydrofluoric acid which attacks glass most violently; yet if we melt glass, it becomes a conductor of electricity and—as Faraday observed—capable of forming an electric combination. Dr. Chase himself is aware of this fact; at the end of the article he says, "gutta-percha fillings being non-conductors, form no battery (!) with dentos." Is "dentos" a conductor of electricity? Most certainly not! Dentos being a mixture of carbonate and phosphate of lime with organic substance is when dry as complete an insulator as ivory; by becoming moist, not the insulating substance becomes a conductor, but the liquid filling the pores conducts the current, the porous cells of Grove's battery are dry insulators as well as moist, yet they let pass the electricity through their pores when these are filled with moisture, acid, etc., without becoming electrical elements themselves. On page 78, Dr. Chase uses the term "strong" What is strong, the electromotive force, (Volts,) or the intensity of the current? (Webers.) As Dr. Chase uses the term "battery," wrong, he should state exactly what he means by strong in an exact scientific sense.

To enter into all his little inaccuracies and logical sommersaults, would be an unnecessary loss of time and not help our object. He gives the coefficients of conductive power:

| | | |
|---|---|---|
| Gold, 60. | While they are: | Gold, 60. |
| Tin, 30. | | Tin, 15. |
| Amalgam, 10. | | Amalgam, (?) |

Did Dr. Chase determine the conductive power of Amalgam, for no text book gives any value of it, and it cannot be supposed that he puts figures at random in a scientific essay?

Gold and "dentos" a battery! if this combination gives electricity then gold and glass, gold and gutta-percha, nay, any combination will give a current, if the liquid is so chosen, that it attacks one of the two substances; Dr. Chase would really have solved the all-vexing question to obtain cheap electricity, e. g. copper, muriatic acid and marble would fill all requirements, and yet such batteries are not used! How does Dr. Chase come to the figures of "strength of batteries" on page 79? They are so fantastical that they are above criticism.

But the worst comes now, and gives me the chief cause to enter into polemics with a well known dentist. Dr. Chase speaks of his prepared cubes, and how they were filled with gold, amalgam, etc.; and exposed to the action of vinegar, and then he gives the loss in each case after one week's immersion.

Who, Doctor, made these experiments? There are only three suppositions as to how such results could have been obtained: that the experiments never were actually made, that they were as chemists say, monstrously "doctored;" that the experimenter was very unskilled and inexperienced in such kind of work, and that his apparatus was insufficient. In face of the well known character of Dr. Chase, the first two suppositions have to be abandoned.

Scientific experiments must be reported, so that every one can repeat them who has proper instruments and skill. Now, there is a great deficiency in Dr. Chase's statements. What is the exact strength of your acid? What is the absolute weight of your cubes? It is necessary to give it, since you give the absolute loss. What was the amount of liquid used? Are you sure that your cubes were made from teeth comporting themselves identically to the acid? Did you dry your cubes equally before and after the immersion in the acid? Did you never use a cube twice? It is necessary to ask these questions, since a careful investigation of the subject has shown me, how important it is to observe every possible precaution in experiments of this kind; perhaps in answering questions, Dr. Chase himself will find the source of the incredible and faulty results which he obtained.

As far as the scanty report of Dr. Chase enabled me to repeat his experiments, I have done this with all the precaution which many years study and practice have taught me, and have come to the following results:

1. Experiments with plugged cubes are absolutely incomparable with each other, if not verified and compared with the results obtained with an unfilled cube of the very same tooth.

2. Different teeth and cubes made thereof lose very differently in the same acid, in the same time, without any visible difference in their structure; e. g. 6 teeth lost in 500 ccm of acetic acid (vinegar,) of 2.1 p. c. strength respectively 13.5, 17.5, 25.3, 12.8, 18.9, 14.4 p. c., the loss included almost no organic substance, but only the inorganic salts, so that a tooth or cube of a tooth never can be used twice.

3. That the drying before and after immersion has to be done very carefully in a water bath, the balance should give milligrams, ($\frac{1}{60}$ grains.)

As I think that a full report of my experiments is not of sufficient interest to all the readers of the "Independent Practitioner," I only give the results; the exact report of the experiments is to every one's disposition if required.

I accompanied every root plugged by a reliable dentist of this city respectively with gold, amalgam, gutta-percha, oxychloride, with an other root of the same tooth, so as to see plainly what the filled root would have lost if not filled; both roots were of nearly the same weight. I brought both roots to equal surfaces exposed to the acid by filling the cavity of the test root with some wax which by previous experiments I had found to be absolutely without influence on the solubility of the tooth; all the four plugged roots together with the four test roots were put into the same vessel, covered with 500 ccm acid of the above strength and left entirely undisturbed for one week; the average weight of an unplugged root was about 300 milligrams and the average absolute loss about 18 p. c. Supposing Dr. Chase's cubes of about 300 milligrams, (the absolute figure matters little here, since the relative loss in comparison with a wax-filled root is the chief point,) his cubes lost:

| | |
|---|---|
| Gold filled cube, - - - - - | 20 p. c. |
| Amalgam " - - - - - - | 13 p. c |
| Gutta-percha " - - - - - | 3 p. c. |
| Wax filled " - - - - - - | 3 p. c. |
| Oxychloride " - - - - - | – p. c. |

Taking the wax-filled cube as the point of comparison, we find the following excesses of loss above the otherwise filled cubes, as obtained by Dr. Chase compared with my experiments.

| Dr. Chase's experiments: | | My experiments: |
|---|---|---|
| Gold filled cube | + 17 p. c. | – 0.9 p. c. |
| Amalgam " | + 10 p. c. | + 1.9 p. c. |
| Gutta-percha " | – p. c. | + 1.3 p. c. |
| Oxychloride " | – 3 p. c. | – 1.9 p. c. |

Every one who understands how to read from experimental figures sees that in my experiments there appears nothing like a law; the figures show, that the filling, except with oxychloride has no effect on the solubility of "dentos," the differences are merely accidental, due to structural difference, only in the case of the root plugged with oxchloride I could find a connection between the smaller loss, though even in this case the total difference was but 1.9 per cent; the oxychloride neutralizing the acid in the immediate neighborhood of the tooth, hereby indirectly protected the tooth; I even could collect about 12 mgr. of acetate of zinc, crytallized near the tooth. Dr. Chase has a most remarkable natural curiosity, more astonishing and valuable to chemists than the great diamond, a tooth which after being simply plugged with oxchloride lost nothing by one week's immersion in his acid in which a gold plugged tooth lost 6 cgr!! The root thus plugged in my experiments lost fully 12.5 per cent. absolute weight! Here, doctor, is an enormous experimental discrepancy. Though Dr. Chase doubtless has done great service to dental science by directing the attention of truth seeking dentists to this very important subject, yet his hypothesis based on wrong notions of electricity and on absolutely erroneous "facts" and faulty experiments has to be abandoned.

I shall not attempt to show the weakness of the "battery" theory by the numerous dental facts that can be brought against it, I only wish to draw attention to a single one. If the theory of "batteries" in teeth was right, the simple conclusion would be that in *all* cases a gold filling would lead to a rapid decay of the so filled tooth, yet dentists assure me that thousands of people who have their decaying teeth plugged with gold never suffer from consequent decay, and that even after having for 30–50 years, a "battery" in a tooth, this tooth is as sound around the gold filling as on other places. Is this true, doctor, and how does it agree with your theory?

It is an undeniable fact, that often a gold filling does not arrest secondary decay when other fillings do, yet I think, that physiology, chemistry and mechanical forces are fully sufficient to explain this fact without having recourse to chimerical batteries. That under special circumstances galvanism may be generated in the mouth by f. i. different metallic fillings, contact of different metals, thermoelectricity etc., is very well possible, yet the chemical action arising from such conditions will be so infinitely small—though the effect on a nerve might be quite considerable—that it would be of no account compared with the large amount of chemical action brought about by the different liquids in the mouth, tooth and pulp.

